# Extracellular Matrix Signalling and Injury Susceptibility: *ACAN* and *FMOD* Variants in Sports-Related Musculoskeletal Injuries

**DOI:** 10.3390/genes17040475

**Published:** 2026-04-17

**Authors:** Agata Rzeszutko-Bełzowska, Agata Leońska-Duniec

**Affiliations:** 1 Faculty of Physical Culture Sciences, College of Medical Sciences, University of Rzeszow, 35-326 Rzeszow, Poland; arzeszutko@ur.edu.pl; 2 Faculty of Physical Education, Gdansk University of Physical Education and Sport, 80-336 Gdansk, Poland

**Keywords:** sports genetics, ACAN, FMOD, anterior cruciate ligament (ACL), musculoskeletal injury susceptibility, extracellular matrix (ECM), proteoglycans, genetic polymorphism

## Abstract

**Background/Objectives**: Musculoskeletal soft-tissue injuries are common among physically active individuals and arise from complex interactions between environmental and biological factors. Genetic variation in genes involved in extracellular matrix (ECM) organization may contribute to individual susceptibility to such injuries. This study investigated whether polymorphisms in aggrecan (*ACAN*, rs2351491 and rs1042631) and fibromodulin (*FMOD*, rs7543148) genes are associated with susceptibility to sports-related injuries. **Methods**: The study included 335 physically active Caucasians, comprising 202 participants with a history of non-contact sports-related musculoskeletal injuries and 133 uninjured controls. Genotyping was performed using real-time polymerase chain reaction. **Results**: No significant associations were observed between the analyzed polymorphisms and overall injury occurrence after correction for multiple comparisons. A nominal association was observed for *ACAN* rs2351491 in the overall injury comparison under the overdominant model (*p* = 0.0457), where CT heterozygotes were more frequent among injured participants. The *ACAN* rs1042631 variant showed nominal associations with anterior cruciate ligament (ACL) injury under the codominant (*p* = 0.0179), recessive (*p* = 0.0243), and overdominant (*p* = 0.0346) models, with the TT genotype associated with lower odds of ACL injury under the recessive model (OR = 0.15, 95% CI: 0.02–1.22). No significant associations were observed for *FMOD* rs7543148 or for haplotype analysis of *ACAN* variants. **Conclusions**: No robust associations were identified between the investigated variants and susceptibility to musculoskeletal soft-tissue injury after correction for multiple testing. Nominal signals observed for *ACAN* variants, particularly in ACL-focused analyses, warrant further investigation but should be interpreted cautiously and confirmed in larger, independent cohorts.

## 1. Introduction

Musculoskeletal soft-tissue injuries are among the most common health problems affecting physically active individuals and professional athletes. These injuries, including muscle strains, ligament ruptures, and tendon injuries, arise from complex interactions between extrinsic and intrinsic biological factors. Although environmental risk factors, such as playing surface, footwear, type of sport, competition level, insufficient warm-up, fatigue, or training load, intensity, and frequency, are well established, growing evidence indicates that inter-individual variability in injury susceptibility is also influenced by genetic determinants [1,2,3,4].

In recent years, both candidate gene studies and genome-wide approaches have identified genetic variants associated with predisposition to musculoskeletal injuries. These polymorphisms are typically located in genes encoding structural proteins of connective and skeletal muscle tissues, enzymes involved in extracellular matrix (ECM) remodeling, and regulators of metabolism, inflammation, vascular function, and cellular signalling [3,5,6,7]. The ECM plays a central role in maintaining the structural integrity and biomechanical properties of ligaments, tendons, and skeletal muscle [8]. It consists of a complex network of structural proteins, proteoglycans, and glycoproteins that regulate collagen fibrillogenesis, tissue hydration, and cell–matrix interactions, thereby contributing to the tensile strength, elasticity, and load distribution of connective tissues exposed to repetitive mechanical stress [8,9]. Disruption of ECM organization or alterations in the expression of its structural components may impair tissue resilience and increase susceptibility to mechanical damage and musculoskeletal injuries [6].

Among the key ECM proteoglycans involved in connective tissue organization is aggrecan, encoded by the *ACAN* gene. Aggrecan is a large chondroitin sulfate proteoglycan that interacts with hyaluronan and other ECM components, forming highly hydrated aggregates that provide resistance to compressive forces in cartilage and other load-bearing tissues [10]. Genetic variation in *ACAN* has been associated with differences in cartilage structure, skeletal development, and predisposition to musculoskeletal disorders, highlighting its importance in maintaining connective tissue homeostasis [11].

Another important ECM component is fibromodulin, encoded by the *FMOD* gene. Fibromodulin belongs to the family of small leucine-rich proteoglycans (SLRPs) that regulate collagen fibrillogenesis and matrix assembly. This protein binds to collagen fibrils and modulates their organization and mechanical properties, thereby contributing to the structural stability of ligaments and tendons [12]. Experimental studies have demonstrated that fibromodulin deficiency results in abnormal collagen fibril morphology and altered biomechanical properties of connective tissues [13,14]. Together, these findings indicate that genetic variation in genes encoding ECM proteoglycans may influence the structural and functional properties of connective tissues, thereby contributing to individual differences in risk of musculoskeletal injuries.

Despite increasing interest in the genetics of sports-related injuries, most studies have focused primarily on structural components of connective tissue, particularly single-nucleotide polymorphisms (SNPs) in collagen-encoding genes [15,16,17,18]. While these studies have provided valuable insights into the genetic architecture of susceptibility to musculoskeletal injuries, considerably less attention has been paid to genes involved in the biological processes that mediate musculoskeletal tissue responses to mechanical stress. In particular, the potential role of genetic variation within the *ACAN* and *FMOD* genes in modulating predisposition to soft-tissue injuries remains largely unexplored. These genes encode ECM proteoglycans that participate in key mechanisms regulating matrix organization, cell–matrix signalling, and tissue responses to mechanical loading [5]. Moreover, much of the existing literature has focused primarily on anterior cruciate ligament (ACL) rupture, whereas other injury phenotypes, including muscle injuries, fractures, dislocations, sprains, and patterns of repeated injury, remain very poorly characterized from a genetic perspective [3,5]. A better understanding of the genetic variability may therefore provide new insights into the mechanisms underlying inter-individual differences in injury risk among physically active individuals. It may contribute to the development of more personalized strategies for injury prevention and rehabilitation.

Accordingly, the present study extends musculoskeletal injury genetics beyond the predominantly studied collagen-related candidates by focusing on ECM proteoglycan genes and broadening the phenotypic framework to encompass diverse injury types and their frequencies. Therefore, the present study aimed to investigate the association between three polymorphisms in the *ACAN* (rs2351491 and rs1042631) and *FMOD* (rs7543148) genes and susceptibility to musculoskeletal soft-tissue injuries in physically active individuals. We hypothesized that genetic variation in these genes may influence connective tissue properties and repair processes, thereby contributing to inter-individual differences in injury risk.

## 2. Materials and Methods

### 2.1. Participants

The study protocol was approved by the Bioethics Committee at the District Medical Chamber in Gdańsk (No. KB-8/22) and conducted in accordance with the Declaration of Helsinki and the Strengthening the Reporting of Genetic Association Studies (STREGA) guidelines.

The study included 335 unrelated physically active Caucasian individuals. The case group consisted of 202 participants with a self-reported history of non-contact sports-related musculoskeletal injuries, whereas the control group included 133 individuals without a history of such injuries. Basic demographic and anthropometric characteristics of the participants are presented in Table 1. Participants were classified as physically active based on their responses to the International Physical Activity Questionnaire (IPAQ). Individuals performing at least 150 min of moderate-intensity physical activity per week or 75 min of vigorous activity were eligible for inclusion.

The majority of participants were involved in endurance-type sports, particularly long-distance running. Among the injured individuals, 142 cases of non-contact muscle injuries were recorded, most frequently affecting the thigh, foot, calf, upper limb girdle, and hip regions. Additionally, 60 participants reported ACL rupture, while 57 individuals experienced other injuries, including fractures, dislocations, or sprains. Because several participants reported more than one injury type, the injured group was additionally stratified by injury count into a single-injury subgroup (*n* = 26) and a multiple-injury subgroup (*n* = 116).

### 2.2. DNA Analyses

Genomic DNA was obtained from buccal epithelial cells collected using sterile swabs (Copan FLOQSwabs, Murrieta, CA, USA). The extraction procedure was performed using the High Pure PCR Template Preparation Kit (Roche, Basel, Switzerland) according to the manufacturer’s guidelines. Genotyping of the selected single nucleotide polymorphisms (SNPs), including *ACAN* rs2351491 (Assay ID: C__25474736_10), *ACAN* rs1042631 (Assay ID: C___8722250_1), and *FMOD* rs7543148 (Assay ID: C___9702305_10), was conducted using TaqMan^®^ SNP Genotyping Assays (Applied Biosystems, Waltham, MA, USA). The real-time polymerase chain reactions (PCRs) were performed on a C1000 Touch Thermal Cycler (Bio-Rad Laboratories, Hercules, CA, USA). Each sample was genotyped in duplicate to ensure accuracy. The reaction mixture contained TaqPath™ ProAmp™ Master Mix (Thermo Fisher Scientific, Waltham, MA, USA), assay mix, genomic DNA template, and nuclease-free water. PCR amplification was performed under standard cycling conditions, including an initial denaturation step followed by 40 amplification cycles. Genotype discrimination and allele calling were performed using CFX Maestro 4.0 Software (Bio-Rad Laboratories, Hercules, CA, USA).

### 2.3. Statistical Analysis

All analyses were conducted in R (v. 4.5.6). Before analyzing polymorphisms, the control group was compared with the study group on additional factors that could interact with genetic variants (age, sex, body weight, height, BMI), referred to as covariates. Numerical covariates were compared using Student’s *t*-test, while the categorical variable (sex) was assessed with the chi-square test. As sex was significantly differentially distributed between groups, it has been used as a covariate in all association models. Information on variant positions, IDs, consequences, and frequencies in the non-Finnish European population has been downloaded from the Genome Aggregation Database (GnomAD) v. 4.1.0. Minor allele frequencies are reported for each case.

The association and interaction analyses of polymorphisms and haplotypes with injury occurrence, as well as the analysis of variant frequencies and compliance with Hardy–Weinberg Equilibrium (HWE), were conducted using the SNPassoc library (version 2.1-0). Each variant was first analyzed for association with the remaining covariates using a generalized linear model (GLM) adjusted for sex. Sex was included in all models, and other covariates were tested for associations (adjusted *p* < 0.05) with any of the polymorphisms. No significant SNP–covariate associations were found (all adjusted *p* > 0.05), and no additional covariate adjustments were conducted.

Associations of polymorphisms were investigated using GLM. Because the underlying mode of inheritance of the investigated variants was unknown, associations were evaluated under several standard genetic models (codominant, dominant, recessive, overdominant, and additive) to explore different possible genotype–phenotype relationships. At the same time, we acknowledge that the use of multiple models increases the multiple-testing burden and supports cautious interpretation of nominal findings. Haplotype analysis was performed for variants within *ACAN* using the haplo.stats library and consisted of two stages. The first stage involved identifying the most probable haplotypes using the expectation–maximization (EM) algorithm. The second stage involved testing the association of each haplotype with injury occurrence using a GLM.

In each association analysis, both nominal *p*-values and *p*-values adjusted for multiple comparisons using the Bonferroni correction are reported.

## 3. Results

### 3.1. Investigated SNPs and Their Characteristics

General characteristics of the polymorphisms are provided in Table 2. The frequencies of the analyzed SNPs, provided for the minor allele in each of the studied subgroups, along with a comparison with values from the population database (GnomAD), are presented in Table S1.

Each variant was also analyzed for HWE (Table S2). All three SNPs showed no significant deviation from HWE in most subgroups. The only deviation was observed for rs1042631 in the control group (*p* = 0.03), with observed genotype counts of CC = 76, CT = 37, and TT = 12, compared with expected counts under HWE of CC = 71, CT = 46, and TT = 8. This deviation reflects an excess of homozygotes and a deficit of heterozygotes relative to expectations. However, this departure was modest and did not reach significance in the full sample (*p* = 0.10) or in any other subgroup. Because all samples were genotyped in duplicate and no systematic deviations were observed for the remaining variants, this modest departure was not regarded as definitive evidence of genotyping error, although its presence should be taken into account when interpreting the results.

### 3.2. Associations of Selected SNPs with All Injuries

Each SNP was analyzed for association with injuries (any injury vs. control group). After adjustment, none of the SNPs were significantly differently distributed between the tested groups under any of the models (Table 3). *ACAN* rs2351491 was nominally (*p* = 0.0457) associated with any injury in the overdominant model, where CT heterozygotes were more likely to be found in the injured group.

### 3.3. Associations of Selected SNPs with the Number and Type of Muscle Injuries

**Figure 1:**
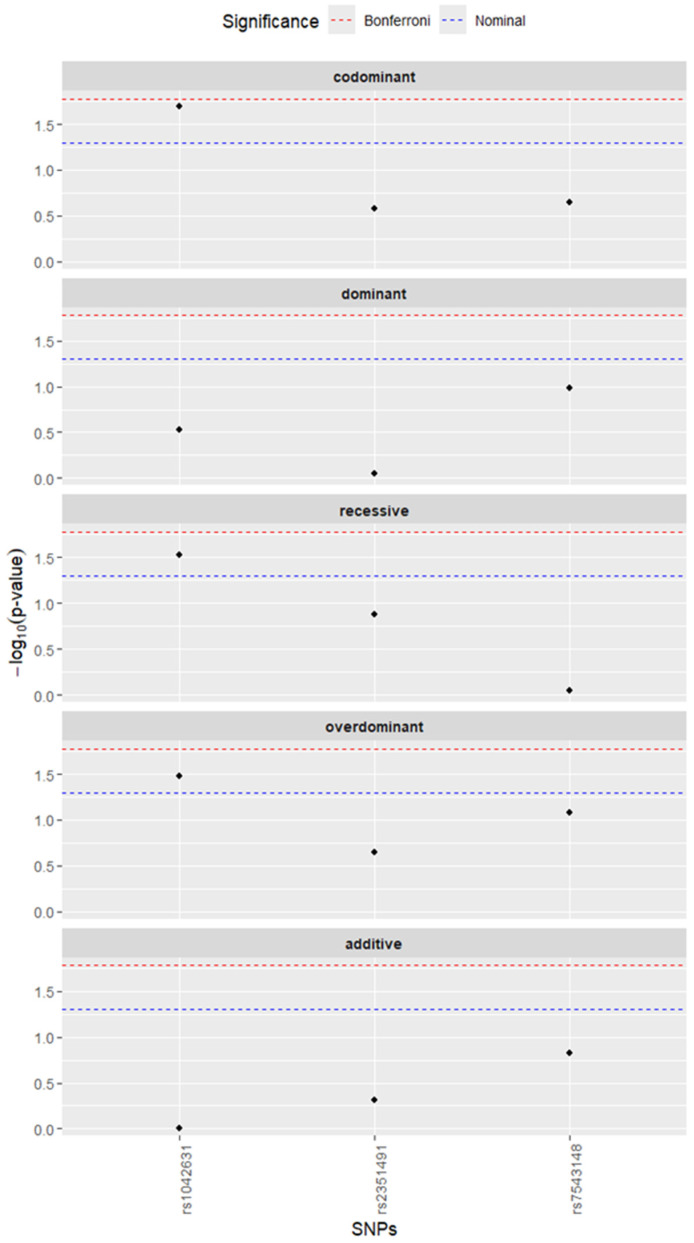
Association of tested SNPs with ACL injury. Participants in the ACL group were compared with the remaining participants in the study. Black dots represent negative logarithms of base ten from *p*-values for each of the tests. Blue line shows nominal *p*-value threshold (0.05), while the red dashed line shows Bonferroni-adjusted *p*-value threshold. Each row on the graph shows results for the model described above.

A detailed analysis of each SNP associated with injuries in different comparisons has also been performed. With regard to the number of injuries (Table S3), no statistically significant associations were found for any of the three SNPs, either in the comparison of the control group vs. participants with more than one injury, or in the comparison of participants with one or no injuries vs. those with more than one injury (all Bonferroni-corrected *p* > 0.05). For different injury types (Table 4), the most notable findings concerned *ACAN* rs1042631 and ACL injury (Figure 1). In the comparison of the control group versus ACL injury, rs1042631 showed nominally significant associations under the codominant (*p* = 0.018), recessive (*p* = 0.024, OR = 0.15, 95% CI: 0.02–1.22), and overdominant (*p* = 0.035, OR = 2.02, 95% CI: 1.05–3.87) models. Similarly, when all other participants were compared with the ACL group, rs1042631 showed nominally significant associations under the codominant (*p* = 0.020), recessive (*p* = 0.029, OR = 0.18, 95% CI: 0.02–1.34), and overdominant (*p* = 0.033, OR = 1.89, 95% CI: 1.06–3.40) models. However, none of these associations remained statistically significant after Bonferroni correction (all corrected *p* > 0.05). No significant associations with ACL, muscle, or other injury types were observed for rs2351491 or rs7543148. Table 4 presents the association results for the rs1042631 polymorphism, while the full set of results for the other SNPs analyzed is provided in Table S4.

### 3.4. Haplotype Analysis

Haplotype analysis of the two *ACAN* variants (rs2351491 and rs1042631) did not reveal significant associations with overall injury occurrence or ACL injury risk across any comparisons (Table 5). Because all estimated effects were close to null and none were statistically significant, the haplotype results did not provide additional support beyond the single-variant analyses. The TT haplotype was not observed in the study sample.

## 4. Discussion

The present study investigated the association between three polymorphisms located in the *ACAN* (rs2351491 and rs1042631) and *FMOD* (rs7543148) genes and susceptibility to sports-related musculoskeletal soft-tissue injuries in physically active individuals. The main finding of this study is that no association remained significant after correction for multiple testing, indicating that the present data do not provide robust evidence of an association between the investigated variants and susceptibility to musculoskeletal injury in this cohort. However, a nominal association was observed for *ACAN* rs2351491 under the overdominant model in the overall injury comparison, with CT heterozygotes more frequent among injured participants. Further comparisons between individuals with multiple injuries and those with either no injury or a single injury showed no statistically significant effects across any of the tested genetic models. In contrast, exploratory analyses stratified by injury type suggested nominal associations of the *ACAN* rs1042631 polymorphism with ACL injury. In comparisons between the ACL-injured group and both uninjured controls and all remaining participants, rs1042631 showed nominal associations across several genetic models, including codominant, recessive, and overdominant. Although the TT genotype was associated with lower odds of ACL injury under the recessive model, this finding should be interpreted with caution because it was based on a small subgroup, was accompanied by wide confidence intervals, depended on the genetic model used, and did not remain significant after Bonferroni correction. No comparable associations were detected for muscle injuries or other injury types. Finally, haplotype analysis of the two *ACAN* variants (rs2351491 and rs1042631) did not reveal significant associations with overall injury occurrence or ACL injury risk. The lack of haplotype associations suggests that the examined variants do not exert a detectable combined genetic effect within the *ACAN* locus in the present cohort. Taken together, these findings suggest that although the analyzed polymorphisms in *ACAN* and *FMOD* do not show strong associations with overall predisposition to sports-related musculoskeletal injuries, nominal signals observed within the *ACAN* locus, particularly for rs1042631 in ACL injury comparisons, may indicate a potential role of ECM proteoglycans in ligament injury susceptibility and warrant further investigation in larger cohorts.

From a biological perspective, *ACAN* is a plausible candidate gene in musculoskeletal injury research, as aggrecan is a key ECM proteoglycan involved in tissue hydration and structural organization in load-bearing connective tissues [10]. By forming large aggregates with hyaluronan and other ECM components, aggrecan contributes to the biomechanical properties of cartilage and other connective tissues exposed to repetitive mechanical stress. Genetic variation within the *ACAN* gene may therefore influence the composition and mechanical behaviour of the ECM, potentially affecting the ability of musculoskeletal tissues to withstand mechanical loading and microdamage. Previous studies have also demonstrated that alterations in aggrecan structure or expression may affect skeletal development and cartilage organization, further supporting the potential relevance of this gene in musculoskeletal tissue homeostasis [11,19]. The present findings may be discussed in the context of previous studies on the *ACAN* locus and musculoskeletal phenotypes, as well as ligament injury risk. In a case–control study of ACL rupture, Mannion et al. [20] reported significant associations within the *ACAN* locus, including an independent association for rs1516797 and haplotype effects involving the rs2351491–rs1042631–rs1516797 region, suggesting that sequence variation within *ACAN* may contribute to susceptibility to ACL injury. In the present study, although none of the associations remained significant after correction for multiple testing, nominal signals were observed for *ACAN* variants, including rs2351491 in the overall injury comparison and rs1042631 across several genetic models in the ACL comparisons. These observations may therefore be interpreted as partially consistent with previous findings implicating variation within the *ACAN* locus in musculoskeletal injury risk. However, they do not confirm a strong independent effect of the examined variants. One possible explanation is that these variants may be in linkage disequilibrium with another functional variant within the *ACAN* locus, suggesting that rs1042631 could serve as a marker for nearby sequence variation that affects ECM composition or connective tissue mechanical properties.

Fibromodulin, encoded by *FMOD*, is a small leucine-rich proteoglycan that regulates collagen fibrillogenesis and ECM organization, processes that are essential for the mechanical stability of ligaments and tendons. [12]. Experimental studies have shown that fibromodulin deficiency leads to abnormal collagen fibril morphology and altered biomechanical properties of connective tissues, indicating an important role of this proteoglycan in maintaining ECM structure and function [13,14,21]. In addition, recent genetic association research has suggested a possible association between the *FMOD* rs7543148 polymorphism and ACL rupture risk, with differences in genotype distribution and a higher frequency of the C allele reported among ACL-injured individuals compared with controls [7]. However, no such association was observed in the present cohort. This discrepancy may reflect differences in sample size, population characteristics, phenotype definition, or the relatively small effect sizes typically observed for individual genetic variants that influence susceptibility to musculoskeletal injury. Overall, while experimental and genetic studies suggest that *FMOD* is a biologically plausible candidate gene for ligament injury risk, the available evidence remains limited and requires confirmation in larger and independent cohorts.

Several limitations of the present study should be acknowledged. Although the cohort size was comparable to that used in previous candidate gene association studies on musculoskeletal injuries [2,22], the present study’s statistical power was limited primarily to detecting moderate-to-large genetic effects, given the sample size, the number of subgroup comparisons, and the evaluation of several genetic models. Accordingly, smaller effects may have remained undetected, and some nominal findings may represent unstable signals. In addition, the study used a candidate-gene approach. It included a limited number of polymorphisms, whereas predisposition to musculoskeletal injuries is likely influenced by multiple genetic variants acting together within complex biological pathways [23]. Another limitation is that injury history was based on self-reported information, which may have introduced both recall bias and phenotype misclassification. This is particularly relevant in genetic association studies, as non-differential misclassification of injury status or injury subtype may attenuate true associations and reduce the ability to detect modest genetic effects. In addition, the inclusion of broader injury categories may have increased phenotype heterogeneity, especially in the overall injury analyses, which could have further diluted any variant-specific effects. Finally, musculoskeletal injury risk is determined not only by genetic predisposition but also by environmental and training-related factors, including training load, biomechanics, and recovery strategies, which were not fully controlled in the present analysis [24]. The absence of detailed biomechanical, postural, and neuromuscular characterization may have further limited the definition of the phenotype, particularly because loading modality and muscle activation patterns can influence muscle activation and tissue loading [25,26,27]. Moreover, the sex distribution in the cohort was uneven, with a predominance of male participants, reflecting the voluntary nature of recruitment and the characteristics of the physically active population that responded to the study invitation. This imbalance may limit the generalizability of the findings, particularly to female athletes. This imbalance represents not only a limitation in terms of generalizability but also a potential source of residual confounding, particularly for ACL injury, for which sex-related differences in risk are well recognized. Although sex was included as a covariate in all association models, such adjustment may not have fully accounted for sex-related differences in injury susceptibility. Therefore, future studies should involve larger, well-characterized cohorts and apply genome-wide or polygenic approaches to better capture the complex genetic architecture underlying susceptibility to musculoskeletal injuries [2,28]. Moreover, integrating epigenetic approaches, including analyses of DNA methylation and other regulatory mechanisms responsive to mechanical load, recovery, training, and supplements, may help elucidate gene–environment interactions that contribute to individual injury risk [29,30].

## 5. Conclusions

The present study investigated the association between polymorphisms in the *ACAN* (rs2351491 and rs1042631) and *FMOD* (rs7543148) genes and predisposition to sports-related musculoskeletal soft-tissue injuries in physically active individuals. No significant associations were observed for overall injury occurrence or injury multiplicity after correction for multiple testing. A nominal association was observed for *ACAN* rs2351491 in the overall injury comparison, and nominal associations for *ACAN* rs1042631 were detected in ACL-focused analyses, suggesting a potential role of these SNPs in sport-related injury risk. However, these findings should be interpreted with caution, particularly given the limited statistical power, the small ACL subgroup, the wide confidence intervals, and the model-dependent nature of the observed signals. No associations were detected for the *FMOD* rs7543148 variant. Thus, the present findings should be regarded as exploratory and do not provide conclusive evidence that the variants investigated contribute to susceptibility to musculoskeletal injury. Further studies in larger and independent cohorts, as well as analyses incorporating additional genetic variants and polygenic approaches, are needed to clarify the contribution of ECM-related genes to the risk of sports-related musculoskeletal injuries.

## Figures and Tables

**Table 1:** Demographic and anthropometric characteristics of the participants.

Characteristic	Controls (*n* = 133)	Injured Participants (*n* = 202)
Males, *n* (%)	86 (64.7%)	159 (78.7%)
Females, *n* (%)	47 (35.3%)	43 (21.3%)
Age (years), mean	36.90	36.54
Height (cm), mean	173.53	175.36
Body mass (kg), mean	71.65	74.00
BMI ^1^ (kg/m^2^), mean	23.37	25.07

^1^ BMI—body mass index.

**Table 2:** Characteristics of the analyzed polymorphisms.

RsID	GnomAD (GRCh38)	Chromosome	Gene	Variant Type/Localisation	CADD Score
rs7543148	1-203348034-C-T	1	*FMOD*	intronic	6.74
rs2351491	15-88854874-C-T	15	*ACAN*	synonymous	8.18
rs1042631	15-88859008-T-C	15	*ACAN*	synonymous	0.152

The reference and alternative alleles are presented according to the Genome Reference Consortium Human Genome Build 38 (GRCh38); the non-reference allele is not always the minor allele. For each of the variants, the gene, variant type, and Phred-scaled Combined Annotation Dependent Depletion (CADD) scores that indicate deleteriousness are reported.

**Table 3:** Results of association analysis of selected SNPs with injury occurrence.

Model	*p*-Value	Adjusted *p*-Value	Genotype	OR	95% CI
rs2351491					
codominant	0.123	0.369	TT	1.00	ref.
			CT	1.52	0.91–2.53
			CC	0.86	0.45–1.66
dominant	0.280	0.840	TT	1.00	ref.
			CT-CC	1.30	0.81–2.09
recessive	0.204	0.613	TT-CT	1.00	ref.
			CC	0.68	0.37–1.23
overdominant	0.0457	0.137	TT-CC	1.00	ref.
			CT	1.59	1.01–2.53
additive	0.959	1.000	0, 1, 2	1.01	0.73–1.40
rs1042631					
codominant	0.352	1.000	CC	1.00	ref.
			CT	1.27	0.77–2.09
			TT	0.68	0.29–1.60
dominant	0.618	1.000	CC	1.00	ref.
			CT-TT	1.12	0.71–1.79
recessive	0.275	0.825	CC-CT	1.00	ref.
			TT	0.63	0.27–1.44
overdominant	0.250	0.751	CC-TT	1.00	ref.
			CT	1.33	0.82–2.17
additive	0.938	1.000	0, 1, 2	0.99	0.69–1.41
rs7543148					
codominant	0.884	1.000	TT	1.00	ref.
			CT	1.13	0.64–2.00
			CC	0.81	0.13–4.96
dominant	0.723	1.000	TT	1.00	ref.
		1.000	CT-CC	1.10	0.64–1.92
recessive	0.800	1.000	TT-CT	1.00	ref.
		1.000	CC	0.79	0.13–4.83
overdominant	0.658	1.000	TT-CC	1.00	ref.
		1.000	CT	1.14	0.64–2.01
additive	0.801	1.000	0, 1, 2	1.07	0.65–1.76

Groups with over 10 participants were included; *p*-values were adjusted using the Bonferroni method; OR—odds ratio, CI—confidence interval.

**Table 4:** Detailed results of the association analysis of rs1042631 with different types of injuries.

Model	*p*-Value	Adjusted *p*-Value	Genotype	OR	95% CI
ACL injury vs. controls					
codominant	0.0179	0.0538	CC	1.00	ref.
			CT	1.79	0.92–3.46
			TT	0.19	0.02–1.55
dominant	0.321	0.964	CC	1.00	ref.
			CT-TT	1.38	0.73–2.60
recessive	0.0243	0.0729	CC-CT	1.00	ref.
			TT	0.15	0.02–1.22
overdominant	0.0346	0.104	CC-TT	1.00	ref.
			CT	2.02	1.05–3.87
additive	0.957	1.000	0, 1, 2	0.99	0.60–1.63
muscle injury vs. controls					
codominant	0.772	1.000	CC	1.00	ref.
			CT	1.17	0.68–2.01
			TT	0.87	0.37–2.08
dominant	0.722	1.000	CC	1.00	ref.
			CT-TT	1.09	0.66–1.80
recessive	0.660	1.000	CC-CT	1.00	ref.
			TT	0.83	0.35–1.93
overdominant	0.516	1.000	CC-TT	1.00	ref.
			CT	1.19	0.70–2.02
additive	0.942	1.000	0, 1, 2	1.01	0.70–1.47
other injuries vs. controls					
codominant	0.594	1.000	CC	1.00	ref.
			CT	1.34	0.66–2.71
			TT	1.51	0.56–4.12
dominant	0.319	0.958	CC	1.00	ref.
			CT-TT	1.38	0.73–2.63
recessive	0.532	1.000	CC-CT	1.00	ref.
			TT	1.37	0.52–3.60
overdominant	0.528	1.000	CC-TT	1.00	ref.
			CT	1.25	0.63–2.47
additive	0.319	0.957	0, 1, 2	1.26	0.80–1.98
ACL injury vs. all other participants					
codominant	0.0200	0.0600	CC	1.00	ref.
			CT	1.70	0.94–3.07
			TT	0.22	0.03–1.69
dominant	0.294	0.883	CC	1.00	ref.
			CT-TT	1.36	0.77–2.42
recessive	0.0294	0.0881	CC-CT	1.00	ref.
			TT	0.18	0.02–1.34
overdominant	0.0332	0.0997	CC-TT	1.00	ref.
			CT	1.89	1.06–3.40
additive	0.982	1.000	0, 1, 2	1.01	0.64–1.57
muscle injury vs. all other participants					
codominant	0.887	1.000	CC	1.00	ref.
			CT	1.01	0.62–1.63
			TT	1.23	0.53–2.86
dominant	0.848	1.000	CC	1.00	ref.
			CT-TT	1.05	0.66–1.64
recessive	0.624	1.000	CC-CT	1.00	ref.
			TT	1.23	0.54–2.80
overdominant	0.936	1.000	CC-TT	1.00	ref.
			CT	0.98	0.61–1.57
additive	0.723	1.000	0, 1, 2	1.07	0.75–1.51
other injuries vs. all other participants					
codominant	0.203	0.609	CC	1.00	ref.
			CT	1.14	0.61–2.15
			TT	2.42	0.95–6.13
dominant	0.309	0.926	CC	1.00	ref.
			CT-TT	1.35	0.76–2.41
recessive	0.0821	0.246	CC-CT	1.00	ref.
			TT	2.30	0.94–5.65
overdominant	1.000	1.000	CC-TT	1.00	ref.
			CT	1.00	0.54–1.84
additive	0.122	0.367	0, 1, 2	1.41	0.92–2.16

Groups with over 10 participants were included; *p*-values were adjusted using the Bonferroni method; OR—odds ratio, CI—confidence interval.

**Table 5:** Haplotype analysis for the two *ACAN* SNPs.

Haplotype		Frequency	OR	Lower 95% CI	Upper 95% CI	*p*-Value
rs2351491	rs1042631					
association with being in the injured group						
T	C	0.5875	1.00	ref.	ref.	ref.
C	C	0.1688	1.06	0.68	1.68	0.7865
C	T	0.2438	1.01	0.70	1.46	0.9465
T	T	0.0000	-	-	-	-
association with being in the ACL group versus controls						
T	C	0.6007	1.00	ref.	ref.	ref.
C	C	0.1548	0.76	0.38	1.53	0.4387
C	T	0.2445	0.97	0.58	1.61	0.9042
T	T	0.0000	-	-	-	-
association with being in the ACL group versus all other groups						
T	C	0.5876	1.00	ref.	ref.	ref.
C	C	0.1686	0.70	0.38	1.31	0.2672
C	T	0.2438	0.94	0.59	1.49	0.7902
T	T	0.0000	-	-	-	-

Groups with over 10 participants were included: OR—odds ratio, CI—confidence interval.

## Data Availability

The data presented in this study are available on request from the corresponding author. The data are not publicly available due to privacy/ethical restrictions.

## Supplementary Materials

The following supporting information can be downloaded at https://www.mdpi.com/article/10.3390/genes17040475/s1, Table S1. Minor allele frequencies for the study subgroups; Table S2. *p*-values for the Hardy–Weinberg equilibrium tests of the investigated polymorphisms; Table S3. Detailed results of the association analysis of selected SNPs with different numbers of injuries; Table S4. Detailed results of the association analysis of rs7543148 and rs2351491 with different types of injuries.

## Abbreviations

The following abbreviations are used in this manuscript:

ACAN	Aggrecan
ACL	Anterior Cruciate Ligament
BMI	Body Mass Index
CADD	Combined Annotation Dependent Depletion
CI	Confidence Interval
DNA	Deoxyribonucleic Acid
ECM	Extracellular Matrix
EM	Expectation–Maximization
FMOD	Fibromodulin
GLM	Generalized Linear Model
GnomAD	Genome Aggregation Database
GRCh38	Genome Reference Consortium Human Genome Build 38
HWE	Hardy–Weinberg Equilibrium
IPAQ	International Physical Activity Questionnaire
OR	Odds Ratio
*p*-value	Probability value
PCR	Polymerase Chain Reaction
ref.	Reference category
SLRP	Small leucine-rich proteoglycan
SNP	Single Nucleotide Polymorphism
STREGA	Strengthening the Reporting of Genetic Association Studies
