# Supplementary material for: Extracellular Matrix Signalling and Injury Susceptibility: ACAN and FMOD Variants in Sports-Related Musculoskeletal Injuries
Source: Genes (Basel). 2026 Apr 17;17(4):475. doi: 10.3390/genes17040475 (PMC13116978; doi:10.3390/genes17040475)
Supplement: Supplementary file 1 [file genes-17-00475-s001.zip › genes-4227362-supplementary.pdf]

Table S1. Minor allele frequencies for the study subgroups.

RsID	GnomAD european	All participants	Controls	Injured group	One injury	More injuries	ACL injury	Muscle injury	Other injuries	Female participants	Male participants
rs7543148	0.1637	0.117	0.112	0.12	0.117	0.122	0.079	0.135	0.114	0.109	0.12
rs2351491	0.3544	0.412	0.408	0.415	0.386	0.435	0.384	0.429	0.465	0.384	0.423
rs1042631	0.2042	0.245	0.244	0.245	0.241	0.248	0.246	0.248	0.307	0.224	0.252

Table S2. P-values for the Hardy-Weinberg equilibrium tests of the investigated polymorphisms.

RsID	All participants	Controls	Injured group	One injury	More injuries	ACL injury	Muscle injury	Other injuries	Female participants	Male participants
rs7543148	0.78	0.65	0.74	1.00	0.67	0.29	1.00	0.54	0.59	0.34
rs2351491	0.91	0.14	0.31	0.24	0.85	0.27	0.61	0.79	0.50	0.59
rs1042631	0.10	**0.03**	0.70	0.14	0.08	0.15	0.17	0.12	0.76	0.08

Table S3. Detailed results of the association analysis of selected SNPs with different numbers of injuries.

Model	P-value	Adjusted p-value	Genotype	OR	95% CI
rs2351491					
no injuries vs more than one injury					
codominant	0.265	0.794	T/T	1.00	ref.
	0.265	0.794	C/T	1.58	0.89-2.84
	0.265	0.794	C/C	1.10	0.53-2.30
dominant	0.201	0.604	T/T	1.00	ref.
	0.201	0.604	C/T-C/C	1.42	0.83-2.45
recessive	0.625	1.000	T/T-C/T	1.00	ref.
	0.625	1.000	C/C	0.85	0.44-1.64
overdominant	0.107	0.322	T/T-C/C	1.00	ref.
	0.107	0.322	C/T	1.53	0.91-2.58
additive	0.563	1.000	0,1,2	1.11	0.78-1.59
more than one injury vs a single injury or no injuries					

codominant	0.592	1.000	T/T	1.00	ref.
	0.592	1.000	C/T	1.31	0.78-2.19
	0.592	1.000	C/C	1.23	0.62-2.42
dominant	0.314	0.942	T/T	1.00	ref.
	0.314	0.942	C/T-C/C	1.28	0.79-2.10
recessive	0.874	1.000	T/T-C/T	1.00	ref.
	0.874	1.000	C/C	1.05	0.57-1.92
overdominant	0.403	1.000	T/T-C/C	1.00	ref.
	0.403	1.000	C/T	1.22	0.77-1.93
additive	0.443	1.000	0,1,2	1.14	0.82-1.58
rs1042631					
no injuries vs more than one injury					
codominant	0.946	1.000	C/C	1.00	ref.
	0.946	1.000	C/T	1.10	0.62-1.95
	0.946	1.000	T/T	0.97	0.40-2.38
dominant	0.818	1.000	C/C	1.00	ref.
	0.818	1.000	C/T-T/T	1.06	0.63-1.80
recessive	0.898	1.000	C/C-C/T	1.00	ref.
	0.898	1.000	T/T	0.94	0.39-2.26
overdominant	0.742	1.000	C/C-T/T	1.00	ref.
	0.742	1.000	C/T	1.10	0.63-1.93
additive	0.911	1.000	0,1,2	1.02	0.69-1.51
more than one injury vs a single injury or no injuries					
codominant	0.587	1.000	C/C	1.00	ref.
	0.587	1.000	C/T	0.84	0.51-1.39
	0.587	1.000	T/T	1.31	0.56-3.06
dominant	0.712	1.000	C/C	1.00	ref.
	0.712	1.000	C/T-T/T	0.92	0.57-1.46
recessive	0.441	1.000	C/C-C/T	1.00	ref.
	0.441	1.000	T/T	1.39	0.60-3.19
overdominant	0.405	1.000	C/C-T/T	1.00	ref.
	0.405	1.000	C/T	0.81	0.49-1.33
additive	0.965	1.000	0,1,2	1.01	0.70-1.45
rs7543148					
no injuries vs more than one injury					
codominant	0.924	1.000	T/T	1.00	ref.
	0.924	1.000	C/T	1.13	0.59-2.16
	0.924	1.000	C/C	0.89	0.12-6.49

dominant	0.744	1.000	T/T	1.00	ref.
	0.744	1.000	C/T-C/C	1.11	0.59-2.07
recessive	0.889	1.000	T/T-C/T	1.00	ref.
	0.889	1.000	C/C	0.87	0.12-6.31
overdominant	0.703	1.000	T/T-C/C	1.00	ref.
	0.703	1.000	C/T	1.13	0.60-2.16
additive	0.801	1.000	0,1,2	1.07	0.61-1.88
more than one injury vs a single injury or no injuries					
codominant	0.965	1.000	T/T	1.00	ref.
	0.965	1.000	C/T	1.08	0.61-1.92
	0.965	1.000	C/C	1.03	0.17-6.29
dominant	0.793	1.000	T/T	1.00	ref.
	0.793	1.000	C/T-C/C	1.08	0.62-1.88
recessive	0.991	1.000	T/T-C/T	1.00	ref.
	0.991	1.000	C/C	1.01	0.17-6.17
overdominant	0.789	1.000	T/T-C/C	1.00	ref.
	0.789	1.000	C/T	1.08	0.61-1.91
additive	0.811	1.000	0,1,2	1.06	0.65-1.75

Groups with over 10 participants were included; P-values were adjusted using the Bonferroni method; OR - odds ratio, CI - confidence interval

Table S4. Detailed results of the association analysis of rs7543148 and rs2351491 with different types of injuries.

Model	P-value	Adjusted p-value	Genotype	OR	95% CI
rs2351491					
ACL injury vs controls					
codominant	0.0932	0.280	TT	1.00	ref.
			CT	1.57	0.78-3.16
			CC	0.56	0.20-1.61
dominant	0.551	1.000	TT	1.00	ref.
			CT-CC	1.22	0.63-2.37
recessive	0.0769	0.231	TT-CT	1.00	ref.
			CC	0.44	0.17-1.15
overdominant	0.0601	0.180	TT-CC	1.00	ref.
			CT	1.85	0.97-3.53
additive	0.615	1.000	0,1,2	0.89	0.57-1.40
muscle injury vs controls					

codominant	0.211	0.634	TT	1.00	ref.
			CT	1.56	0.90-2.70
			CC	1.00	0.50-2.03
dominant	0.226	0.677	TT	1.00	ref.
			CT-CC	1.37	0.82-2.30
recessive	0.437	1.000	TT-CT	1.00	ref.
			CC	0.78	0.42-1.46
overdominant	0.0779	0.234	TT-CC	1.00	ref.
			CT	1.56	0.95-2.55
additive	0.698	1.000	0,1,2	1.07	0.76-1.51
other injuries vs controls					
codominant	0.506	1.000	TT	1.00	ref.
			CT	1.54	0.74-3.21
			CC	1.35	0.56-3.25
dominant	0.261	0.783	TT	1.00	ref.
			CT-CC	1.47	0.75-2.91
recessive	0.883	1.000	TT-CT	1.00	ref.
			CC	1.06	0.49-2.29
overdominant	0.336	1.000	TT-CC	1.00	ref.
			CT	1.37	0.72-2.61
additive	0.424	1.000	0,1,2	1.19	0.78-1.82
ACL injury vs all other participants					
codominant	0.263	0.790	TT	1.00	ref.
			CT	1.22	0.65-2.30
			CC	0.59	0.22-1.56
dominant	0.900	1.000	TT	1.00	ref.
			CT-CC	1.04	0.57-1.91
recessive	0.131	0.394	TT-CT	1.00	ref.
			CC	0.52	0.21-1.28
overdominant	0.228	0.683	TT-CC	1.00	ref.
			CT	1.43	0.80-2.55
additive	0.488	1.000	0,1,2	0.86	0.57-1.31
muscle injury vs all other participants					
codominant	0.449	1.000	TT	1.00	ref.
			CT	1.38	0.84-2.28
			CC	1.22	0.63-2.37
dominant	0.228	0.685	TT	1.00	ref.
			CT-CC	1.34	0.83-2.15

recessive	0.973	1.000	TT-CT	1.00	ref.
			CC	1.01	0.56-1.82
overdominant	0.263	0.789	TT-CC	1.00	ref.
			CT	1.29	0.82-2.02
additive	0.403	1.000	0,1,2	1.15	0.83-1.58
other injuries vs all other participants					
codominant	0.481	1.000	TT	1.00	ref.
			CT	1.18	0.61-2.29
			CC	1.66	0.74-3.73
dominant	0.402	1.000	TT	1.00	ref.
			CT-CC	1.30	0.70-2.43
recessive	0.268	0.805	TT-CT	1.00	ref.
			CC	1.50	0.74-3.04
overdominant	0.941	1.000	TT-CC	1.00	ref.
			CT	0.98	0.55-1.74
additive	0.240	0.721	0,1,2	1.28	0.85-1.92
rs7543148					
ACL injury vs controls					
codominant	0.449	1.000	TT	1.00	ref.
			CT	0.57	0.23-1.41
			CC	0.87	0.08-9.94
dominant	0.221	0.664	TT	1.00	ref.
			CT-CC	0.59	0.25-1.41
recessive	0.976	1.000	TT-CT	1.00	ref.
			CC	0.96	0.08-10.94
overdominant	0.207	0.622	TT-CC	1.00	ref.
			CT	0.57	0.23-1.41
additive	0.277	0.830	0,1,2	0.66	0.31-1.43
muscle injury vs controls					
codominant	0.560	1.000	TT	1.00	ref.
			CT	1.37	0.75-2.49
			CC	0.77	0.11-5.59
dominant	0.355	1.000	TT	1.00	ref.
			CT-CC	1.31	0.73-2.35
recessive	0.744	1.000	TT-CT	1.00	ref.
			CC	0.72	0.10-5.21
overdominant	0.296	0.888	TT-CC	1.00	ref.
			CT	1.37	0.76-2.50

additive	0.451	1.000	0,1,2	1.23	0.72-2.08
other injuries vs controls					
codominant	0.990	1.000	TT	1.00	ref.
			CT	0.96	0.43-2.15
			CC	0.87	0.08-9.93
dominant	0.904	1.000	TT	1.00	ref.
			CT-CC	0.95	0.44-2.08
recessive	0.916	1.000	TT-CT	1.00	ref.
			CC	0.88	0.08-9.95
overdominant	0.929	1.000	TT-CC	1.00	ref.
			CT	0.96	0.43-2.16
additive	0.891	1.000	0,1,2	0.95	0.48-1.90
ACL injury vs all other participants					
codominant	0.225	0.674	TT	1.00	ref.
			CT	0.50	0.21-1.16
			CC	1.02	0.11-9.40
dominant	0.103	0.308	TT	1.00	ref.
			CT-CC	0.53	0.24-1.18
recessive	0.905	1.000	TT-CT	1.00	ref.
			CC	1.15	0.12-10.53
overdominant	0.0840	0.252	TT-CC	1.00	ref.
			CT	0.50	0.21-1.16
additive	0.150	0.450	0,1,2	0.60	0.29-1.25
muscle injury vs all other participants					
codominant	0.275	0.824	TT	1.00	ref.
			CT	1.56	0.90-2.72
			CC	0.85	0.14-5.18
dominant	0.142	0.426	TT	1.00	ref.
			CT-CC	1.50	0.87-2.56
recessive	0.779	1.000	TT-CT	1.00	ref.
			CC	0.77	0.13-4.72
overdominant	0.110	0.331	TT-CC	1.00	ref.
			CT	1.57	0.90-2.72
additive	0.213	0.640	0,1,2	1.36	0.84-2.21
other injuries vs all other participants					
codominant	0.987	1.000	TT	1.00	ref.
			CT	0.94	0.46-1.95
			CC	1.01	0.11-9.30

dominant	0.879	1.000	TT	1.00	ref.
			CT-CC	0.95	0.47-1.91
recessive	0.985	1.000	TT-CT	1.00	ref.
			CC	1.02	0.11-9.37
overdominant	0.870	1.000	TT-CC	1.00	ref.
			CT	0.94	0.46-1.94
additive	0.896	1.000	0,1,2	0.96	0.51-1.80

Groups with over 10 participants were included; P-values were adjusted using the Bonferroni method; OR - odds ratio, CI - confidence interval
